# Pericyte Expression of VEGF-A Minimally Impacts Ocular Vascular Development and Neovascularization

**DOI:** 10.3390/cells14181473

**Published:** 2025-09-21

**Authors:** Yong-Seok Song, Shoujian Wang, Samay Inampudi, Hope Risa, Christine M. Sorenson, Nader Sheibani

**Affiliations:** 1 Department of Ophthalmology and Visual Sciences, University of Wisconsin School of Medicine and Public Health, Madison, WI 53705, USA; song224@wisc.edu (Y.-S.S.); shoujianwang@wisc.edu (S.W.); risa@wisc.edu (H.R.); 2 McPherson Eye Research Institute, University of Wisconsin School of Medicine and Public Health, Madison, WI 53705, USA; 3 Department of Pediatrics, University of Wisconsin School of Medicine and Public Health, Madison, WI 53705, USA; inampudi2@wisc.edu; 4 Department of Cell and Regenerative Biology, University of Wisconsin School of Medicine and Public Health, Madison, WI 53705, USA

**Keywords:** PDGF, VEGFA, pericytes, endothelial cells, retinal vasculature, oxygen-induced ischemic retinopathy, choroidal neovascularization

## Abstract

Pericytes produce vascular endothelial growth factor-A (VEGF-A; hereafter referred to as VEGF). VEGF inhibits pericyte proliferation and migration through enhanced VEGFR2 and PDGFRβ heterodimerization. Heterodimerization of these receptors on perivascular supporting cells, mediated by VEGF in culture, mitigates signaling through these receptors and promotes a quiescent phenotype. However, the detailed cellular mechanisms and the significance of these interactions in vivo require further investigation. The cell-autonomous activities of pericyte VEGF expression during vascular development and neovascularization remain unknown. Here we utilized mice conditionally lacking *Vegfa* in pericytes (*Vegfa*^PC^) to examine its impact on retinal vascular development and pathological ocular neovascularization. Vascular integrity was also assessed in older mice using fundus imaging and fluorescein angiography. The lack of *Vegfa* pericyte expression delayed the initial spreading of the superficial layer of the retinal vasculature. Mice lacking *Vegfa* pericyte expression had similar numbers of retinal endothelial cells and arteries to their wild-type littermates. However, the number of pericytes was significantly reduced in younger *Vegfa*^PC^ mice but increased in more mature mice. In addition, pericyte *Vegfa* deficiency did not impact responses during oxygen-induced ischemic retinopathy and laser-induced choroidal neovascularization. Thus, pericyte VEGF expression plays a role during early stages of retinal vascular development with limited influence on mature retinal vascularization, its integrity, and neovascularization.

## 1. Introduction

Vascular endothelial growth factor-A (VEGF-A, hereafter referred to as VEGF) is a key modulator of vascular development and vascular homeostasis functions [1,2]. Its altered production in response to hypoxia/ischemia contributes to various pathologies including cancer and various eye diseases [3,4]. The altered expression of VEGF is recognized as a key mediator of ocular neovascularization. VEGF is utilized as a major target for treatment of ocular diseases with a neovascular component [3]. These ocular diseases include retinopathy of prematurity, diabetic retinopathy, and age-related macular degeneration. Delineating the intracellular mechanisms leading to altered VEGF expression and activity in these diseases has been the subject of numerous studies including retinal vascular development and neovascularization.

Many cell types produce VEGF, which affects various cellular functions including vascular permeability through interactions with its receptor [5,6]. The mouse retinal vasculature, which develops postnatally, has provided a great opportunity to learn more about the cellular sources of VEGF. These cells respond to VEGF in various ways, impacting developmental and homeostatic processes of the retinal vasculature. Many studies have shown that cellular constituents of the retinal neurovasculature produce VEGF [7,8,9,10,11,12]. These cells respond to VEGF in a paracrine or autocrine fashion, including retinal perivascular supporting cells [11,13], collectively referred to as mural cells, pericytes, and smooth muscle cells.

Pericytes act as vascular stabilizers. Through this function they aid in angiogenesis, inflammation, blood flow regulation, permeability modulation, and tissue maintenance. Although their ratio to endothelial cells varies in each tissue vascular bed, their balanced interaction is essential for proper vascular function and homeostasis. The human retinal vasculature has a pericyte-to-endothelial-cell ratio of 1:1, suggesting a critical need for pericytes in this vasculature [14,15]. Although the significance of this high pericyte density in the retinal vascular bed is unknown, when disrupted, pathogenesis ensues as observed in diabetic retinopathy. Loss of pericytes in the retinal vasculature has long been recognized as an early hallmark of diabetic retinopathy.

Pericyte migration and proliferation, as well as recruitment and vessel stability, are influenced by growth factors including VEGF [16]. VEGF induces B-cell lymphoma-2 (Bcl-2) expression, a prosurvival factor, and vice versa [17,18]. Both VEGF and Bcl-2 influence not only apoptosis but also cell proliferation, adhesion, and migration [19,20,21]. Mouse retinal pericytes secrete over 4- to 6-fold higher VEGF compared with retinal endothelial cells in culture [22,23]. However, an increased level of VEGF expression is generally associated with retinal and choroidal pathologies such as retinopathy of prematurity and neovascular age-related macular degeneration (nAMD), perhaps due to disrupting normal pericyte functions and maturation [24]. Although pericyte loss is associated with vascular instability, the impact that the loss of VEGF expression in pericytes has on their recruitment, vascular maturation, and stabilization has not, to the best of our knowledge, been previously addressed. 

The cell-autonomous activity of VEGF has been addressed in several cellular constituents of retinal neurovasculature including endothelial cells, astrocytes, microglial cells, retinal pigment epithelium (RPE) cells and neurons, but its cell-autonomous role in pericytes has not been previously evaluated. Although the targeted deletion of VEGF in endothelial cells has no impact on the initial formation of retinal blood vessels, its absence results in endothelial cell loss and degeneration of mature blood vessels [7]. In other cells such as astrocytes, VEGF expression acts as a key promoter of vascular permeability driven by inflammatory mediators [5] and expansion of newly forming blood vessels [25,26]. In myeloid cells, VEGF expression is not essential for retinal neovascularization but instead enhances fibrosis and tumor formation [27,28,29]. In neurons, VEGF deletion impacts function [8,30,31], while VEGFR2 deletion in retinal neurons leads to an aberrant gradient of VEGF and abnormal vascularization [32]. Targeted deletion of VEGF in RPE cells leads to aberrant choriocapillaris formation and vision loss [33]. Thus, aberrant VEGF expression in many retinal neurovascular cell types can lead to vascular dysfunction. How pericyte expression of VEGF impact these normal and pathological processes remain elusive.

Pericytes are recognized as an important regulator of endothelial cell sprouting and branching morphogenesis by modulation of VEGF/VEGFR2 signaling through expression of VEGFR1 [34]. However, the impact of pericyte VEGF expression on ocular vascular development and neovascularization is unknown. Platelet-derived growth factor receptor β (Pdgfrβ) activation on pericytes facilitates their recruitment to the newly forming blood vessels [35,36]. This has made Pdgfrβ a good choice for pericyte-targeted gene disruption. Here, using *Pdgfrb*-Cre transgenic mice, we assessed the impact of the targeted deletion of *Vegfa* in pericytes on postnatal retinal vascularization, integrity, and neovascularization.

## 2. Materials and Methods

### 2.1. Ethics Statement

The experiments performed here were approved by the Institutional Animal Care and Use Committee of the University of Wisconsin School of Medicine and Public Health (IACUC assurance number: D16-00239, approval date: 16 June 2023) and conducted in accordance with the Association for Research in Vision and Ophthalmology Statement for the Use of Animals in Ophthalmic and Vision Research. The euthanasia by CO_2_ asphyxiation was carried out according to approved protocols.

### 2.2. Experimental Animals

Conditional *Vegfa* mice lacking expression in pericytes (*Vegfa^PC^* mice) were generated by crossing mice carrying a conditional *Vegfa* allele (*Vegfa*^flox/flox^; obtained from Genentech, San Francisco, CA, USA, and denoted hereafter as wild-type (WT [1]) with *Pdgfrb*-Cre mice. Pdgfrb-Cre*^45Vli^* mouse generation and utility have been assessed in previous studies by our laboratory and others using various reporter mice [37,38,39]. All mice were from a C57BL/6J background, screened to ensure the absence of Rd8 and Rd1 mutations, and were maintained at the University of Wisconsin animal facilities. Mice of both sexes were used in all the studies conducted. 

Genotyping of the Tg (*Pdgfrb*-Cre)*^45Vli^* mouse line was performed using the following primers: 5′-GCATTTCTGGGGATTGCTTA-3′ and 5′-CCCGGCAAAACAGGTAGTTA-3′ [37,40] and *Vegfa*^flox/flox^ mice with 5′-CCTGGCCCTCAAGTACACCTT-3′ and 5′-TCCTACGACGCATTTCTAG-3′. We crossed *Vegfa*^flox/flox^ mice with Tg (Pdgfrb-Cre)*^45Vli^* mice [38] to obtain mice heterozygous for the floxed allele that expressed Pdgfrb-Cre. These mice were bred and the progeny screened as described above to obtain homozygous mice for the *Vegfa*^flox/flox^ allele that also expressed Cre. The efficiency of this Cre has been previously assessed by us and others using a Tomato reporter mouse and shown to target nearly all the pericytes in the retina [37]. We maintained the colony by crossing mice homozygous for the *Vegfa*^flox/flox^ allele that were Cre-expressing with mice homozygous for the *Vegfa*^flox/flox^ allele and genotyping.

Cre expression was confirmed by immunostaining of retinal flatmounts using anti-CD31 (BD Biosciences, San Diego, CA USA; 553370; 1:50 dilution) to stain the retinal vasculature and anti-Cre (Millipore-Sigma, St. Louis, MO, USA; 69050-3; 1:100 dilution) to stain pericytes expressing Cre at 4 °C overnight. The retinas were incubated with anti-rat-Cy2 for CD31 (Jackson ImmunoResearch, West Grove, PA, USA; 712-225-150; 1:200 dilution) and anti-rabbit Alexa 594 (ThermoFisher Scientific, Carlsbad, CA, USA; A-11037; 1:400 dilution) for 2 h at room temperature. Pericytes were identified by their nuclear morphology and abluminal vascular location. Pericyte identity was further confirmed by co-staining with anti-Cre and anti-CD13, a mouse pericyte-specific marker [15] (R&D Systems, Minneapolis, MN, USA; AF2335). 

### 2.3. Trypsin-Digested Retinal Vessel Preparation

Trypsin digestion of retinal flatmounts is commonly used to assess retinal pericyte and endothelial cell numbers and their ratio. This technique allows a quantitative assessment of retinal vascular cell density and integrity. In preparation, eyes were fixed with 4% paraformaldehyde (at least 24 h), and the retina was removed and washed overnight in distilled water. Retinas were incubated overnight (37 °C) in 3% trypsin solution (Electron Microscopy Sciences, Hatfield, PA USA; 22200, in 0.1 M Tris, 0.1 M maleic acid, pH7.8, containing 0.2 M NaF). The retinal digestion was monitored so that the vasculature remained intact, but the majority of the neural retina was digested away. Following the completion of digestion, the retinas underwent four radial cuts for flattening. Retinas were mounted on glass slides, dried, and used for periodic acid–Schiff (PAS) and hematoxylin staining. Pericytes and endothelial cells were identified by nuclear morphology and their luminal (endothelial cells) and abluminal (pericytes) locations on the retinal capillary networks, and their numbers were determined in a masked fashion by counting four fields of view (100 µm^2^) from four quadrants of each mid-retina zone (×400) [37]. Eyes from at least 5 mice (both male and female) were used for this analysis.

### 2.4. Visualization of Retinal Vasculature

Mouse retinal vascularization begins to form shortly after birth, and a superficial layer of vessels is formed during the first week of life, i.e., by postnatal day 7 (P7). These vessels begin sprouting from the optic nerve at P0 and spread to the periphery following a scaffolding that has been laid out by astrocytes ahead of the spreading vasculature. After reaching the periphery, they sprout deep into the retina to form deep (P14) and intermediate retinal vascular layers (P21) during the second and third weeks of life. Although the formation of the primary vascular plexus of the retina is completed by P21, the retinal vasculature continues to undergo pruning and remodeling, adjusting its oxygen need, and reaches maturation by P42 [41,42,43].

To examine the developing retinal vasculature, enucleated eyes at different postnatal days were fixed in 4% paraformaldehyde (1 h at room temperature) and kept in 100% methanol for a minimum of 24 h at −20 °C [37]. Retinas were incubated in blocking buffer containing 3% bovine serum albumin (BSA, Jackson ImmunoResearch, West Grove, PA, USA; 001-000-162) and 0.3% Triton X-100 in PBS at room temperature for 1 h. Following blocking, samples were incubated with anti-collagen IV (Millipore-Sigma; AB756P; 1:250 dilution) and anti-GFAP (ThermoFisher; 14-9892-82, 1:100 dilution) prepared in blocking buffer at 4 °C overnight. Samples were washed with PBS and incubated with secondary antibodies anti-rabbit-Alexa 594 for anti-collagen IV (ThermoFisher; A-11037; 1:400 dilution) and anti-mouse Alexa 488 for GFAP staining (ThermoFisher; A-11001; 1:400 dilution) prepared in blocking buffer for 2 h at room temperature. After washing with PBS, retinas were mounted on glass slides for fluorescence microscopy. Images were captured in digital format using a fluorescence stereomicroscope (Nikon SMZ25, Nikon, Tokyo, Japan) equipped with a digital camera (Orca Flash 4.0 Lt, C11440, Hamamatsu, Japan).

To assess potential differences in the rate of vessel sprouting, eyes from P5 mice when the initial spreading of retinal vasculature was nearly halfway between the optic nerve and retinal periphery were utilized. This allowed us to determine the rate of spreading, the number of endothelial sprouts and the extension of filopodia, a characteristic that is normally associated with the leading tip cells at the forefront of the spreading vasculature, and the status of the retinal astrocytes. The number of endothelial sprouts and the extension of filopodia were determined using high power fields (×150) with retinal flatmounts. Data are presented as the average and standard deviation of tip cell sprouts counted in at least 6 mice of each genotype using both male and female mice.

To identify major arteries in the developing mouse retina, eyes from P7 mice were collected, and the retinas were stained using anti-alpha smooth muscle actin antibody conjugated with FITC (Millipore-Sigma; F3777; 1:300 dilution) at 4 °C overnight. The numbers of major arteries in mice were counted, and data are presented as the average and standard deviation in at least 12 mice including male and female mice from each genotype.

### 2.5. Fundus Imaging

To assess the impact of VEGF expression on the integrity of mature retinal vasculature, mice (9-month-old male and female individuals) were anesthetized (ketamine 100 mg/kg and xylazine 10 mg/kg) and their pupils dilated (2.5% phenylephrine and 0.5% tropicamide; Bausch and Lomb Inc., Bridgewater, NJ, USA). Mice were maintained on a heating pad for warmth, and their eyes were examined by fundus imaging on a Micron III retinal imaging system (Phoenix Laboratories Inc., Pleasanton, CA, USA). We used equal image depth, the same magnification, and had the circular border of the image distinctly in focus for all images. Fundus images were evaluated for the presence of bright spots. The leakiness of the blood vessels was assessed by fluorescein angiography using the same imaging system equipped with appropriate objective for fluorescein detection. Anesthetized mice received a single intraperitoneal injection of 1% sodium fluorescein (AK-FLOUR, NDC 17478-101-12, Akorn, Lake Forest, IL, USA). Fluorescent fundus photographs were captured 5 min after fluorescein injection and used for assessment of vascular leakiness.

### 2.6. Laser-Induced Choroidal Neovascularization (CNV) and OIR

For laser photocoagulation studies, 12-week-old mice of both genotypes were used (10 mice per group, including both male and female mice). Ketamine hydrochloride (80 mg/kg) and xylazine (10 mg/kg) were used to anesthetize the mice, and a combination of one drop of 2.5% phenylephrine followed by a drop of 0.5% tropicamide was used for pupil dilation. At the posterior pole, each eye received 3-laser photocoagulation (75 µm spot size, 0.1 sec duration, 120 mW) spots at the 9, 12, and 3 o’clock positions using a slit lamp delivery system of an OcuLight GL diode laser (Iridex, Mountain View, CA, USA). A handheld coverslip, as a contact lens, was utilized to view the retina. Eyes were fixed in 4% paraformaldehyde (2 weeks later); the choroid/RPE complex was incubated in blocking buffer (5% fetal calf serum, 20% normal goat serum in PBS) and then incubated with FITC-conjugated Isolectin B4 (Vector Laboratories, Burlingame, CA, USA; FL-1201) diluted 1:100 in PBS (containing 20% fetal calf serum, 20% normal goat serum) at 4 °C overnight. Fluorescence microscopy was used to image slides. Image J software (National Institute of Health, Bethesda, MD; http://rsb.info.nih.gov/ij/; version 1.52, accessed on 20 May 2024) and pixel intensities (in µm^2^) were used to determine the degree of neovascularization.

For OIR, 7-day-old (P7) mice with dams were housed for 5 days (P12) in an atmosphere of 75 ± 0.5% oxygen (incubator temperature 23 ± 2 °C). A PROOX model 110 oxygen controller (BioSpherix LTD; Parish, NY, USA) continuously monitored the oxygen level. The mice were then moved to room air (20% oxygen) for 5 days (P17), and maximum retinal neovascularization was noted [42,44]. To determine the levels of retinal neovascularization, retinal wholemounts were prepared from P17 mice and stained with anti-collagen IV (Millipore-Sigma; AB756P;1:250). Retinal wholemounts were imaged using a fluorescence stereomicroscope (Nikon SMZ25, Nikon, Japan) equipped with a digital camera (Orca Flash 4.0 Lt, C11440, Hamamatsu, Japan). The central vessel obliteration area was quantified in pixels by tracing the edges of the vessel obliteration area using ImageJ Version 1.52 in a masked fashion. The neovascular tuft area was semiautomatically quantified in pixels using the SWIFT_NV ImageJ plugin developed by Stahl et al. [45]. The quantified vessel obliteration area and neovascular tuft areas were normalized by the total retinal area, which was measured by manually outlining the edges of the retina using ImageJ.

### 2.7. RNA Purification and Real-Time qPCR Analysis

To gain insights into the potential regulatory mechanisms impacted by pericyte VEGF expression, we examined the expression of angioregulatory genes in retinas of wild-type and transgenic mice (P7 and P21 reared in room air) by qPCR analysis. Tissue was lysed using Trizol reagent (ThermoFisher; 15596018), and total RNA was extracted with a RNeasy mini kit (Qiagen, Maryland, CA, USA; 74014). Total RNA (1 µg) was used for cDNA synthesis with RNA to cDNA EcoDry Premix (Takara, Mountain View, CA, USA; 639549). For qPCR (cDNA was diluted 1:10), samples were run in triplicate for each biological replicate with a Master cycler Real plex (Eppendorf, Fisher Scientific, Hanover Park, IL USA,) using TB-green advantage qPCR premix (Takara; 39676) with gene-specific primers (Table 1). The mouse ribosomal protein L13a (Rpl13a) was used as the housekeeping control gene. Standard curves were generated from known quantities of each target gene from linearized plasmid. The linear regression line for DNA (ng) was determined with relative fluorescent units (RFU) at a threshold fluorescence value (Ct). Gene targets were quantified from tissue extracts comparing RFU at the Ct to the standard curve. This was normalized by the simultaneous amplification of the housekeeping gene RpL13a.

### 2.8. Data Analysis

Statistical analysis was performed using GraphPad Prism version 8 for Windows software (GraphPad Software, La Jolla, CA, USA). The Shapiro–Wilk test was used for assessing the normal distribution of the data. Student’s unpaired *t*-test (two-tailed) was performed for statistical analysis between two groups. One-way analysis of variance (ANOVA) followed by Tukey’s multiple comparison test was used to determine the significant differences between the means of every possible two groups in all experimental groups. Mean ± standard deviation is shown. *p* < 0.05 was considered significant.

**Table 1:** Primers used for qPCR studies.

Gene	Forward	Reverse
*Vegfa*	5′-GGAGAGCAGAAGTCCCATGA-3′	5′-ACTCCAGGGCTTCATCGTTA-3′
*Vegfb*	5′-TGGTGCCATGGATAGACGTT-3′	5′-TTGTTTGACCACATTGCCCA-3′
*Vegfc*	5′-GTGTGCGAATCGACTGAAGC-3′	5′-GTTCAGATGTGGCCTTTTCCA-3′
*Vegfr1*	5′-GGCCCGGGATATTTATAAGAAC-3′	5′-CCATCCATTTTAGGGGAAGTC-3′
*Vegfr2*	5′-CCCCAAATTCCATTATGACAA-3′	5′-CGGCTCTTTCGCTTACTGTT-3′
*Vegfr3*	5′-CCATCTCAACGTGGTCAACC-3′	5′-AAGTTGGAGAGGTTGCCGTA-3′
*Pdgfb*	5′-CTCGGCCTGTGACTAGAAGT-3′	5′-GGATTCTCACCGTCCGAATG-3′
*Pdgfrb*	5′-ATCGCGCCACCTTAATCAAC-3′	5′-GCTAAGAAGTCCATGCCGTT-3′
*Angpt1*	5′-GTGCAGCAACCAGCGCCGAA-3′	5′-CGCACTCTCACGGCAGTTCCC-3′
*Angpt2*	5′-CCGGTCAGCACCGCTACGTG-3′	5′-ATGCGCCTCGTTGCCTTCCC-3′
*Tek/Tie2*	5′-TCCAACATCACTGACTCCACA-3′	5′-GCCCTGAACCTTATACCGGA-3′
*Acta2/Sma*	5′-GGCATCCACGAAACCACC-3′	5′-CATGGTGGTACCCCCTGAC-3′
*Cspg4/Ng2*	5′-CTCACGAGCCCCTGTATCTC-3′	5′-GGGTGCCCTCTGTACTTCAT-3′
*Mmrn2*	5′-CATCACCGGGTTCCAGTCTA-3′	5′-CACGCTCTCTCACCCTTTTG-3′
*Cd93*	5′-ACAACAGGTCTCTTCGTCCA-3′	5′-GTATGTGCCCAACTCGAACC-3′
*Kc/Cxcl1*	5′-ACAGGGGCGCCTATCGCCAA-3′	5′-CGGTTTGGGTGCAGTGGGGC-3′
*Cxcl11*	5′-TGGCAGAGATCGAGAAAGCT-3′	5′-GCACCTTTGTCGTTTATGAGC-3′
*Cxcr3*	5′-TCTGCTGGTGTTAACTCTTCCA-3′	5′-GTTGATGTTGAACAAGGCGC-3′
*Bcl2*	5′-GGAGAGCGTCAACAGGGAGA-3′	5′-CAGCCAGGAGAAATCAAACAGAG-3′
*Bim*	5′-AGTGTGACAGAGAAGGTGGACAATT-3′	5′-GGGATTACCTTGCGGTTCTGT-3′
*Rpl13a*	5′-TCTCAAGGTTGTTCGGCTGAA-3′	5′-GCCAGACGCCCCAGGTA-3′

## 3. Results

### 3.1. Validation of the Genetic Model for Loss of VEGF in Pericytes

**Figure 1:**
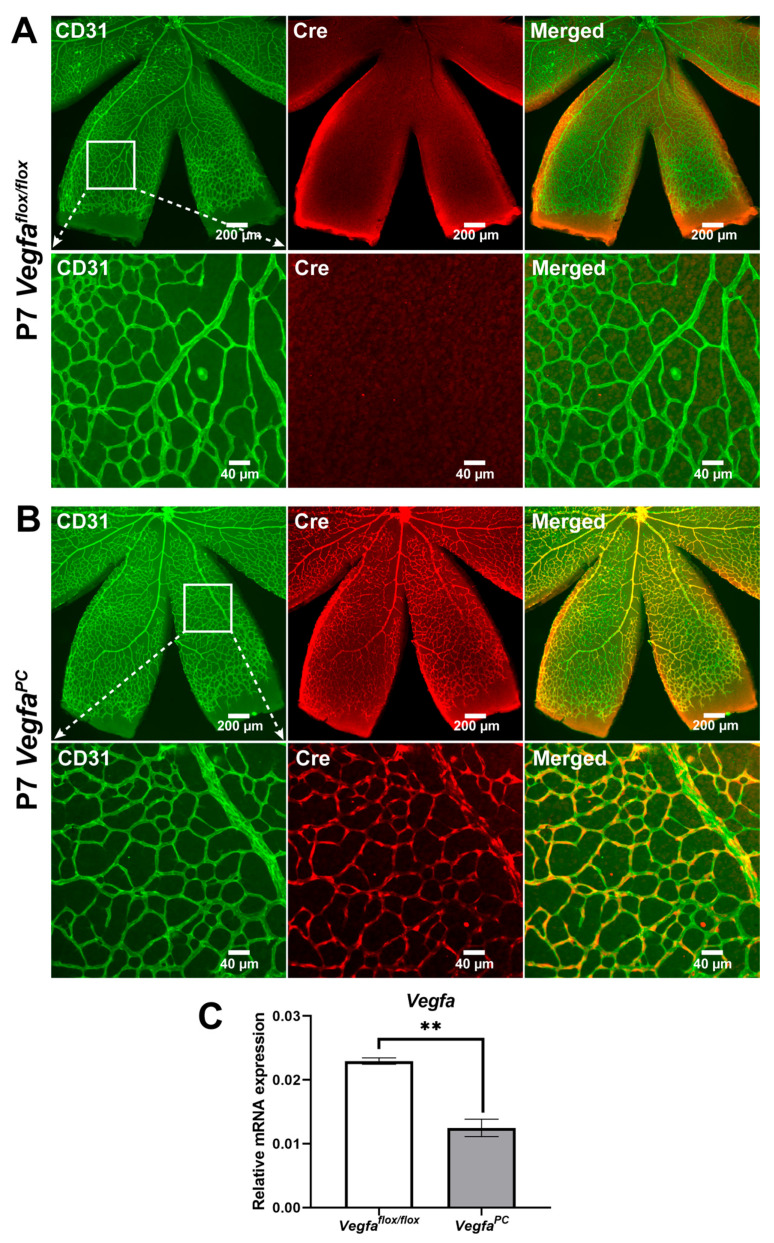
Cre expression in the retinal wholemounts and retinal Vegfa mRNA levels. Retinal wholemounts were prepared from P7 (**A**) *Vegfa*^flox/flox^ and (**B**) *Vegfa*^PC^ mice and stained with anti-CD31 (PECAM-1, an endothelial cell marker) and anti-Cre antibodies. The wholemounts were photographed using a fluorescence microscope with different magnifications (top panels: ×6, bottom panels: ×30). The white box in lower-magnification images indicates the field of view for higher-magnification images. Please note specific Cre-staining of the pericytes, recognizable by their nuclear morphology and vascular localization. (**C**) The expression levels of vascular endothelial growth factor a (*Vegfa*) in the retinas of 9-month-old *Vegfa*^flox/flox^ and *Vegfa*^PC^ mice were assessed by qPCR analysis. The expression level of *Vegfa* was significantly lower in the retinas from *Vegfa*^PC^ mice (*n* = 3, ** *p* < 0.01). The observations in these mice were sex-independent.

Retinal neurovascular cells including retinal endothelial cells, pericytes, astrocytes, retinal ganglion cells, inner retinal neurons, photoreceptors, and retinal pigment epithelium cells all produce VEGF to maintain retinal neurovascular homeostasis through paracrine and autocrine activities. However, the cell-autonomous VEGF function in pericytes remains unknown. Here we utilized mice lacking *Vegfa* expression in pericytes, *Vegfa^PC^* mice, to assess its impact on retinal vascular development, integrity, and ocular neovascularization. Figure 1 shows retinas from P7 *Vegfa*^flox/flox^ and *Vegfa^PC^* mice stained with anti-Cre and anti-CD31 (to visualize retinal vasculature). As indicated at P7 only the superficial layer of retinal vasculature is formed, which simplifies immunostaining and imaging studies of the developing vasculature. In retinas from *Vegfa^PC^* mice (Figure 1B)*,* Cre staining was restricted to nearly all the pericytes, while no Cre staining was noted in retinal endothelial cells or the retinal vasculature from WT (*Vegfa*^flox/flox^) littermates (Figure 1A). These results are similar to our studies with mice carrying a conditional Tomato allele and expressing the same *Pdgfrb*-Cre [40]. This was further confirmed by co-staining of pericytes with anti-Cre and CD13. The whole retinal *Vegfa* (all isoforms) expression in adult mice (at 9 months old, when the retinal vasculature is completely mature and stable) was reduced by nearly 2-fold in *Vegfa^PC^* mice compared to the expression in retinas from *Vegfa*^flox/flox^ mice (Figure 1C). VEGF immunostaining of the whole tissue is challenging since, as indicated, many cells in the retina produce VEGF, a secreted protein, making it difficult to colocalize staining to only pericytes in the vasculature. We have also had minimal success with mouse antibodies available for Western blotting.

### 3.2. Early Retinal Vascularization Was Delayed in Vegfa^PC^ Mice

**Figure 2:**
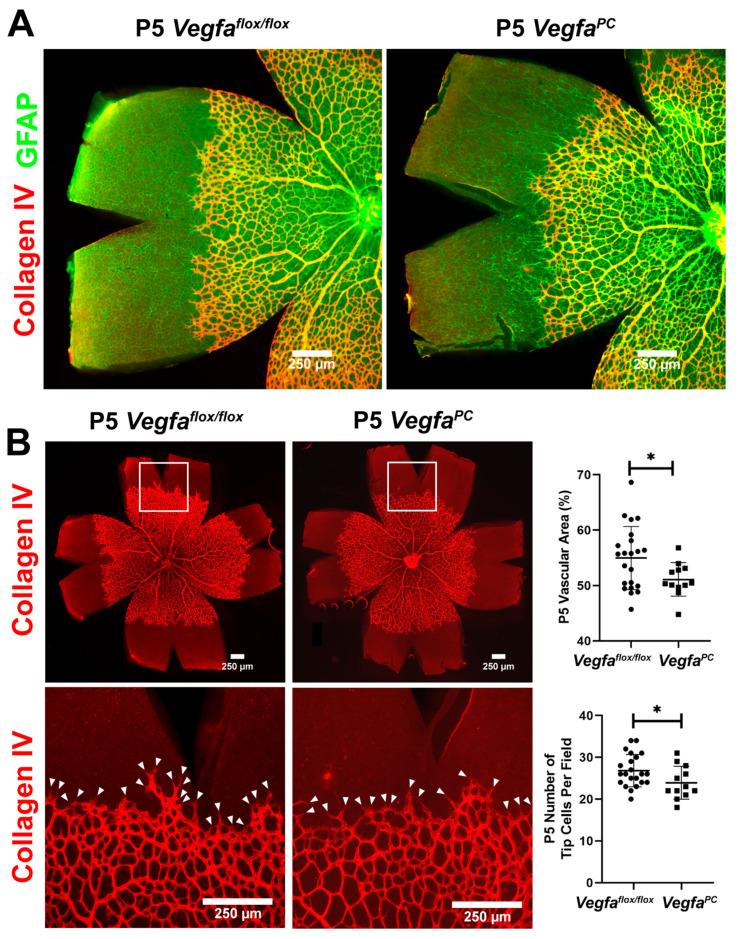
Decreased retinal vasculature expansion and tip cell sprouting in *Vegfa*^PC^ mice. (**A**) Retinal whole mounts from P5 *Vegfa*^flox/flox^ and *Vegfa*^PC^ mice were stained with anti-collagen IV (red) and anti-glial fibrillary acidic protein (GFAP, green) antibodies, and merged images are shown. (**B**) Retinal wholemounts showing staining with anti-collagen IV at different magnifications. The coverage of retinal vasculature relative to the total retinal area was measured using ImageJ and is presented as percentages (*n* > 22, * *p* < 0.05). The mean numbers of tip cell filopodia (arrowheads) per field were determined for each retina as detailed in Materials and Methods (*n* > 24, * *p* < 0.05). The white boxes indicate areas magnified for quantification shown below.

**Figure 3:**
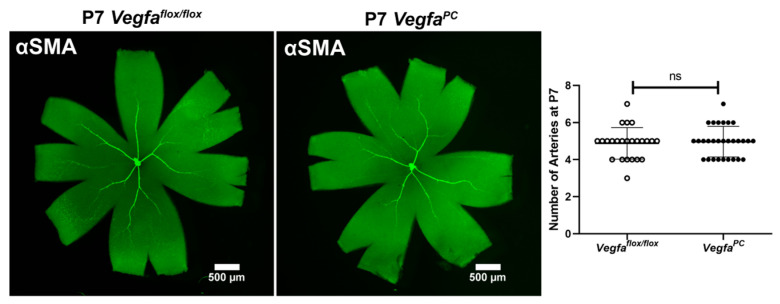
Similar numbers of retinal arteries in *Vegfa^f^*^lox/flox^ and *Vegfa*^PC^ mice. Retinas from P7 *Vegfa*^flox/flox^ and *Vegfa*^PC^ mice were wholemount-stained with anti-α-smooth muscle actin (αSMA) antibody to identify major arteries. Mean numbers of major arteries in both male and female mice were quantified per retina and are shown (*n* > 24, ns: not significant).

Retinal vascular development proceeds after birth in the mouse, with formation of the superficial layer commencing over the first 7 days. The role of *Vegfa* pericyte expression in retinal vascular development and pathological neovascularization is not well delineated. At P5, spreading of the retinal superficial vascular layer and tip cell numbers were determined in wholemount retinas stained with anti-collagen IV (vascular) and anti-GFAP (astrocytes) (Figure 2). At P5, the retinal superficial vascular layer spreading and tip cells are most noticeable as actively vascularizing the retina. The number of endothelial sprouts and the extension of filopodia, a characteristic associated with the leading tip cells at the distal end of sprouts, were significantly reduced in *Vegfa^PC^* mice compared to their *Vegfa*^flox/flox^ littermates. To visualize retinal arteries, retinas from P7 mice were wholemount stained for α-smooth muscle actin (αSMA) to examine the number of major retinal arteries. At this stage the expression of αSMA is restricted to the perivascular smooth muscle cells that cover the larger vessels (mainly arteries). *Vegfa*^flox/flox^ and *Vegfa^PC^* mice of both sexes had similar numbers of arteries (Figure 3).

**Figure 4:**
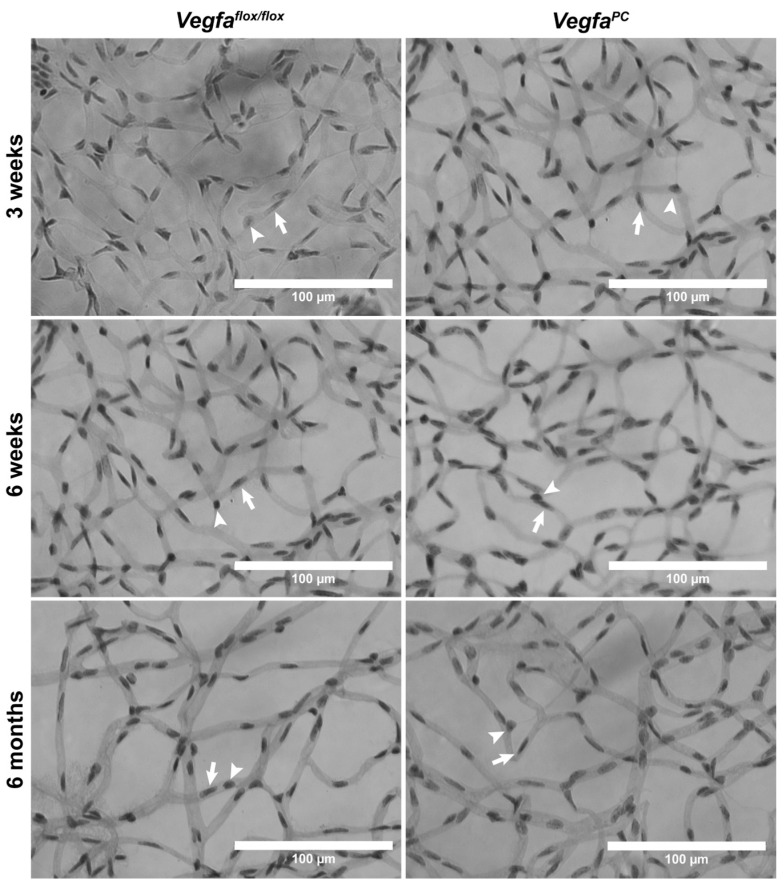
Altered endothelial cell (EC) and pericyte (PC) density in *Vegfa*^PC^ mice. Retinas were isolated from 3-week-old, 6-week-old, and 6-month-old *Vegfa*^flox/flox^ or *Vegfa*^PC^ mice and prepared for trypsin digest followed by HE/PAS staining as detailed in Methods. EC (arrows) and PC (arrow heads) numbers were determined by quantifying the number of cells per high-power field (×400; 100 µm^2^). The quantitative analysis of the data is presented as mean ± standard deviation and summarized in Table 2. Eyes from at least 6 mice of each genotype and sex were used.

To gain additional insights into the density of retinal vascular cells, we utilized wholemount retinal trypsin digests prepared from older mice. Decreased pericyte numbers, due to development or loss, can impact vascular integrity and leakage. Retinal pericyte and endothelial cell numbers were assessed from retinal trypsin digest preparations at 3 weeks of age (when formation of primary vascular plexus is completed), 6 weeks (when pruning and remodeling is completed), and later at 6 months (when the retinal vasculature has completely matured and stabilized; see Figure 4). We next determined the density of retinal pericytes and endothelial cells by counting the number of cells per high-power field (×400; 100 µm^2^) (Table 2). Although the numbers of endothelial cells were not significantly different in 3-week-old mice, the numbers of pericytes were significantly lower in *Vegfa*^PC^ mice. No significant differences were noted in the number of retinal endothelial cells or pericytes in 6-week-old mice. However, at 6 months old, a modest but significant increase in pericyte numbers was seen in *Vegfa*^PC^ mice compared to *Vegfa*^flox/flox^ littermates. No capillary degeneration was noted in *Vegfa*^PC^ mice for any of the ages or sexes examined.

### 3.3. Lack of VEGF in Pericytes Does Not Impact the Integrity and Differentiation Status of Retinal Vasculature 

**Figure 5:**
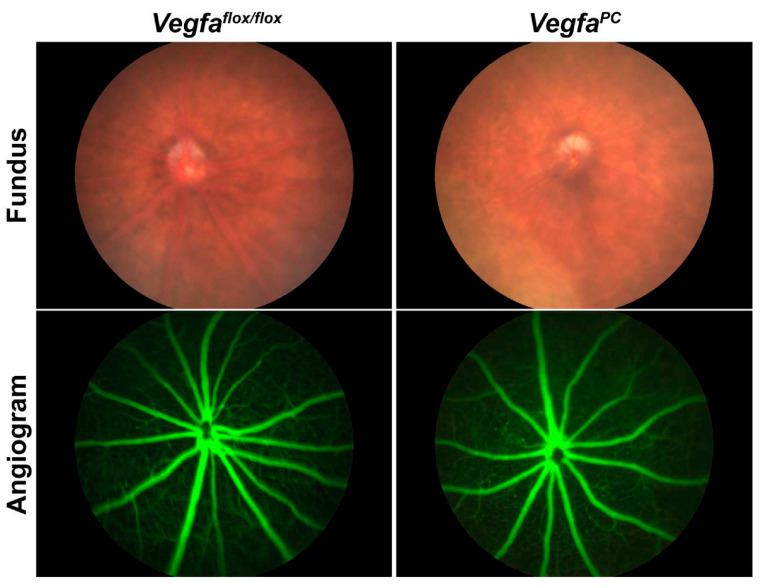
Fundus and fluorescein angiogram images of *Vegfa*^flox/flox^ and *Vegfa*^PC^ mice. Fundus images were taken from 9-month-old mice using a Micron III indirect camera. The mice were subsequently injected with sodium fluorescein 10% solution (100 mg/kg) for the fluorescein angiogram. Please note that no sign of bright spots in fundus images or fluorescein leakage was noted in images from mice of either genotype and no signs of fundus abnormalities (upper panels) or leakiness (lower panels) were noted in these images, either. At least 5 mice of each genotype, including males and females, were examined.

**Figure 6:**
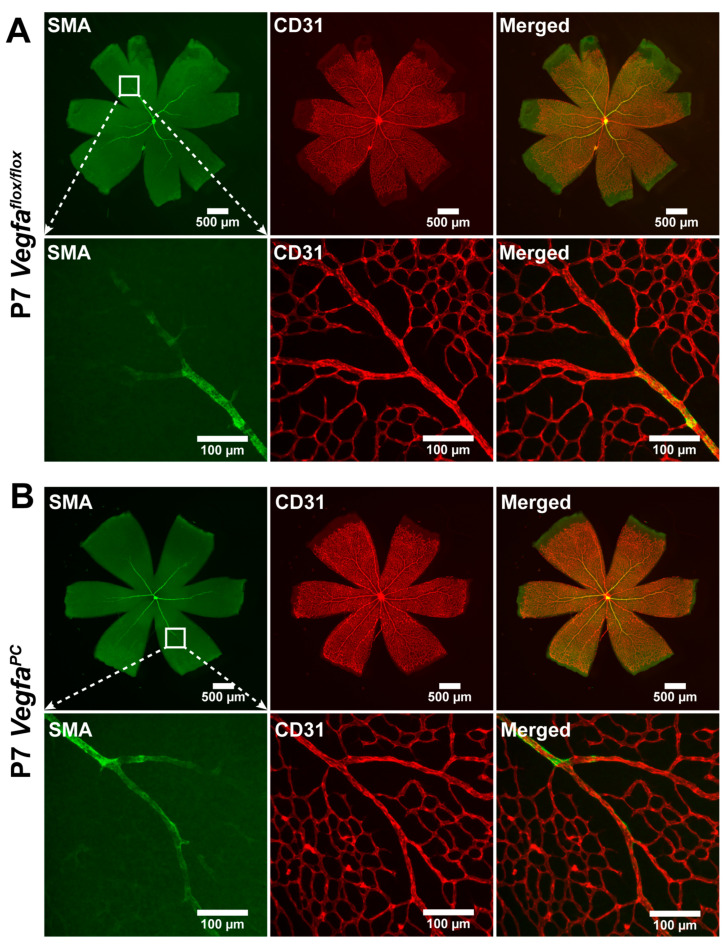
Expression of α-smooth muscle actin (αSMA) and CD31 in the retinal vasculature of *Vegfa*^flox/flox^ and *Vegfa*^PC^ mice. Wholemount retinas were prepared from P7 (**A**) *Vegfa*^flox/flox^ and (**B**) *Vegfa*^PC^ mice and stained with anti-αSMA and anti-CD31 antibodies. The wholemount retinas were imaged using a fluorescent microscope at different magnifications (top panels: ×2.5 and bottom panels: ×30). White boxes in lower-magnification images show the field of view of higher-magnification images. At least 5 mice of each genotype, including males and females, were examined. Please note that αSMA staining is restricted to the major arteries and not capillaries; its expression in capillaries is generally associated with activated pericytes.

Given the propensity of mice lacking endothelial cell VEGF expression to undergo vascular degeneration with age, we next analyzed the retinal vasculature in older mice by fundus imaging and fluorescence angiography (FA). The vascular integrity as assessed by fundus imaging and FA was similar in 9-month-old *Vegfa*^flox/flox^ and *Vefga^PC^* mice (Figure 5). We also examined αSMA expression in the retinal vasculature by wholemount staining of retinas with anti-αSMA and -CD31 (Figure 6). Identical αSMA staining on larger arteries was noted regardless of pericyte VEGF status. No signs of abnormality or leakiness were noted on fundus imaging in *Vegfa*^flox/flox^ or *Vefga^PC^* mice of either sex. 

### 3.4. Lack of VEGF Expression in Pericytes Does Not Affect Area of Vessel Obliteration and Retinal Neovascularization During OIR

**Figure 7:**
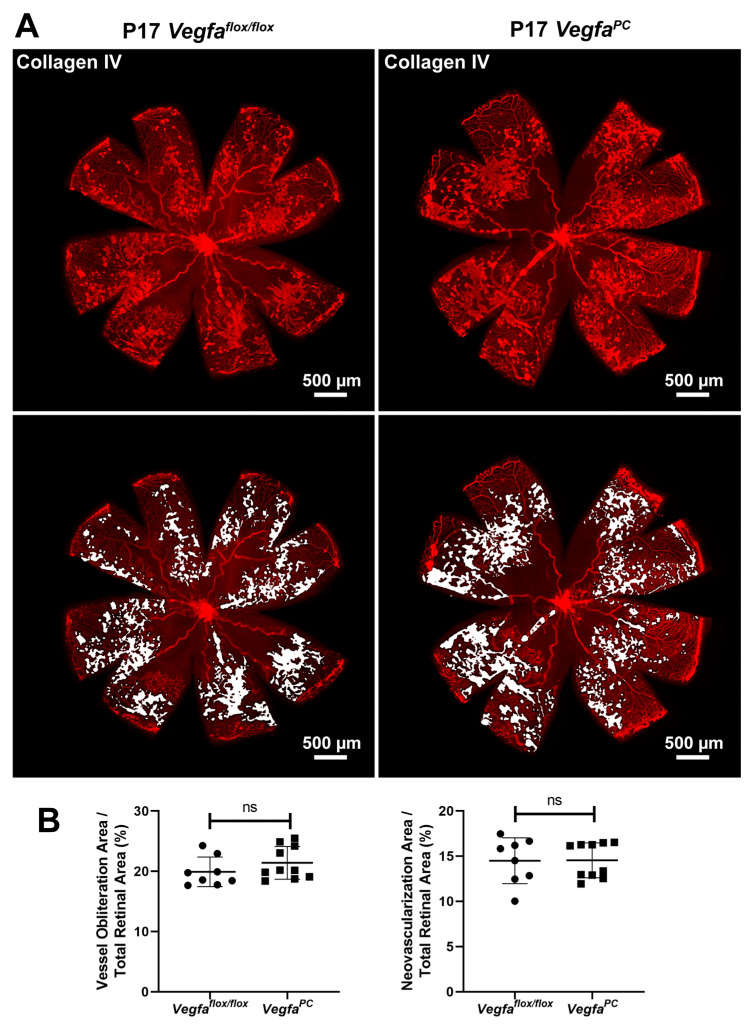
Vessel obliteration and neovascularization during oxygen-induced ischemic retinopathy (OIR) were not impacted in *Vegfa*^PC^ mice. (**A**) P7 *Vegfa*^flox/flox^ and *Vegfa*^PC^ mice were exposed to hyperoxia (75%) for 5 days (P12) and returned to room air for 5 days (P17). Retinas from male and female P17 (when maximum neovascularization is expected) *Vegfa*^flox/flox^ and *Vegfa*^PC^ mice were wholemount-stained with anti-collagen IV, which stains the basement membrane of retinal vasculature. (**B**) The areas of vessel obliteration (left) and neovascularization (right) relative to the whole retinal area were quantitatively analyzed (*n* ≥ 8; each point represents one retina. ns: not significant).

Given other reports showing that a lack of VEGF expression in endothelial cells demonstrated the most obvious effects under stress [39], here we assessed the impact that a lack of pericyte VEGF expression had during oxygen-induced ischemic retinopathy (OIR). *Vegfa^PC^* mice (P7) and their *Vegfa*^flox/flox^ littermates were subjected to hyperoxia (75%) for 5 days then brought to room air for an additional 5 days, as detailed in Materials and Methods. This procedure initially promotes vessel loss and stifles growth of new vessels but later, in room air, the retina notes its ischemic situation and undergoes pathologic neovascularization, giving rise to tortuous leaky vessels (OIR stage 2: P17). Lack of VEGF expression in pericytes did not impact the level of neovascularization or area of vessel obliteration in mice at P17 (Figure 7).

### 3.5. Lack of VEGF Expression in Pericytes Did Not Impact Choroidal Neovascularization

**Figure 8:**
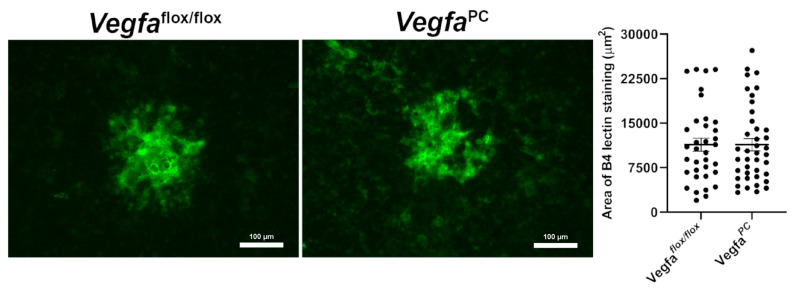
Lack of *Vegfa* in pericytes did not affect choroidal neovascularization. Laser photocoagulation-induced rupture of Bruch’s membrane model was performed using 8-week-old mice. After 14 days, the RPE/choroid was wholemount-stained with Isolectin B4-FITC, and the area of neovascularization was quantified using ImageJ. Eyes from at least 10 mice of each genotype, including males and females, were used.

VEGF is widely considered to be the driving force for CNV. Pericytes secrete VEGF and stabilize the vasculature. Patients with nAMD have increased pericyte numbers and intraocular PDGF-BB levels [46,47]. Here, we assessed the necessity of VEGF expression in pericytes for formation of CNV following laser photocoagulation-induced rupture of Bruch’s membrane, by staining wholemount choroid/RPE with B4 lectin to stain neovascularization. The levels of neovascularization were determined as detailed in Materials and Methods. Figure 8 demonstrates that *Vegfa*^flox/flox^ and *Vefga^PC^* mice exhibited nearly identical levels of neovascularization following laser photocoagulation. Thus, the pericyte VEGF expression did not impact CNV. 

### 3.6. The Impact of Pericyte VEGF Expression on Angioregulatory Gene Expression in the Retina

**Figure 9:**
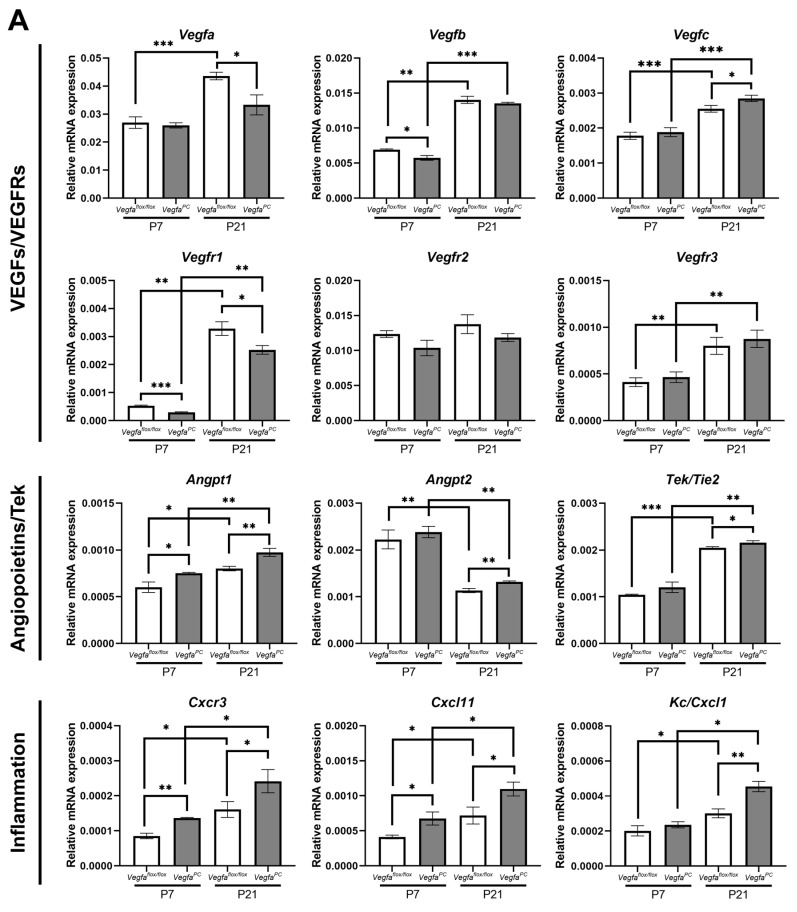

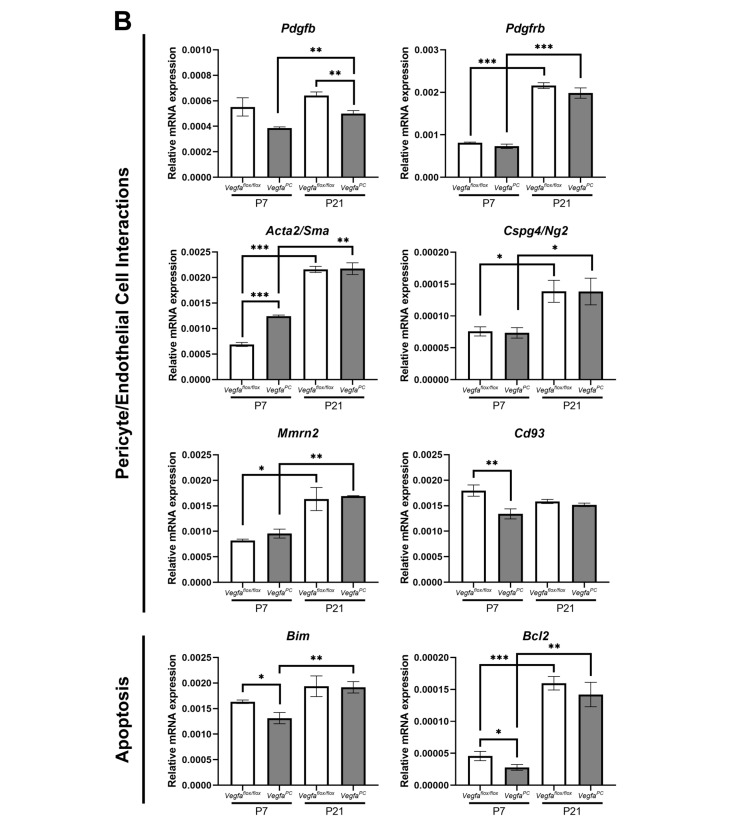
Expression of angioinflammatory mediators in the retinas from *Vegfa*^flox/flox^ and *Vegfa*^PC^ mice. Total RNA was isolated from the retinas obtained from P7 and P21 mice. RT-qPCR was performed to assess the expression levels of (**A**) *Vegfa*, *Vegfb*, *Vegfc*, *Vegfr*1, *Vegfr*2, *Vegfr*3, *Angpt*1, *Angpt*2, *Tek/Tie2*, *Cxcr3*, *Cxcl*11, and *Kc/Cxcl*1 and (**B**) *Pdgfb*, *Pdgfrb*, *Acta2/Sma*, *Cspg4/Ng2*, *Mmrn*2, *Cd*93, *Bim*, and *Bcl*2. Expression levels of the genes were normalized by *Rpl13a* expression. The primer sequences are listed in Table 1. (*n* = 3, * *p* < 0.05, ** *p* < 0.01, *** *p* < 0.001). White bars correspond to Cre^−^ (wild-type control), and gray bars correspond to Cre^+^ (pericyte VEGF-deficient). Changes in the levels of expression are summarized in Table 3. *Vegfa*, vascular endothelial growth factor A; *Vegfr*, vascular endothelial growth factor receptor; *Angpt*, Angiopoietin; *Tek*, TEK receptor tyrosine kinase; *Cxcr*3, C-X-C motif chemokine receptor 3; *Cxcl*, C-X-C motif chemokine ligand; *Acta2/Sma*, actin alpha 2 smooth muscle; *Cspg*4, chondroitin sulfate proteoglycan 4; *Mmrn*2, multimerin 2; *Rpl13a*, ribosomal protein L13A.

Proper expression of VEGF is important for vascular development and integrity. To determine whether the expression of VEGF in pericytes impacts expression of other related angioregulatory genes, we examined the expression of various genes with important roles in angiogenesis and inflammatory processes in the retinas from mice at P7 and P21, when the formation of retinal superficial layer and complete vascular plexus are formed, respectively. Figure 9 shows the expression of various forms of Vegf (Vegfa, Vegfb, and Vegfc), various Vegf receptors (Vegfr1, Vegfr2, and Vegfr3), Pdgfb and Pdgfrb, Angiopoietin-1 (Angpt1), Angiopoietin-2 (Angpt2), and Tek/Tie2, Acta2/αSMC, Cspg4/Ng2, Multimern-2 (Mmrn2), Cd93, Cxcl1, Cxcl11, Cxcr3, Bim, and Bcl-2 in retinas from *Vegfa*^flox/flox^ and *Vegfa*^PC^ mice. The Vegfa is the predominant Vegf form expressed in the retina, followed by the expression of Vegfb and Vegfc at much lower levels. A significant decrease in Vegfa level was noted in retinas from P21 *Vegfa*^PC^ mice, as well as modest changes in Vegfb and Vegfc (Figure 9A and Table 3). Vegfr2 is the predominant receptor expressed in the mouse retina compared to Vegfr1 and Vegfr3. Figure 9A shows a significant decrease in Vegfr1 in retinas from *Vegfa*^PC^ mice. No significant change in the level of Vegfr2 and Vegfr3 was noted in retinas from *Vegfa*^flox/flox^ and *Vegfa*^PC^ mice.

The level of Angpt1 was significantly increased in retinas from P7 and P21 *Vegfa*^PC^ mice compared to their *Vegfa*^flox/flox^ counterpart, and at P21, *Angpt*2 was also increased in retinas from *Vegfa*^PC^ mice (Figure 9A). Interestingly, Angpt2 was the only gene assessed whose expression decreased in retinas from P21 mice compared to P7 regardless of Vegfa status. No significant changes in the expression of Tek/Tie2 were noted in retinas from *Vegfa*^flox/flox^ and *Vegfa*^PC^ mice. The expressions of Cxcl11 and Cxcr3 were significantly increased in retinas from P7 and P21 *Vegfa*^PC^ mice (Figure 9A), while Cxcl1 was only increased in P21 *Vegfa*^PC^ mice compared to *Vegfa*^flox/flox^ mice. However, the expression of Cxcl1 was increased in P21 retinas compared to P7 retinas regardless of Vegfa status.

Although the expression of Pdgfrb was similar in retinas from *Vegfa*^flox/flox^ and *Vegfa*^PC^ mice, the level of Pdgfb was lower in retinas from *Vegfa*^PC^ mice at P21 (Figure 9B). In retinas from P7 *Vegfa*^PC^ mice, a significant increase in Acta2/αSMA expression was noted, although similar levels were observed at P21 (Figure 9B). CD93 and its ligand Mmrn2 were recently shown to be produced by endothelial cells and located in the basement membrane shared by endothelial cells and pericytes [48,49,50]. The disruption of these interactions affects adhesion and migration of activated endothelium and recruitment of pericytes, impacting angiogenesis and vascular stability [51]. This is mediated through modulation of cytokines in both endothelial cells and pericytes, such as sequestration of Vegfa by Mmrn2 [52]. The absence of Mmrn2 results in decreased pericyte coverage of blood vessels [53]. Although we noted a significant increase in the Mmrn2 expression in retinas from P21 mice compared to P7, no significant changes in expression were noted with *Vegfa* deletion from pericytes (Figure 9B). In contrast, the expression of Cd93 was significantly decreased in P7 *Vegfa*^PC^ but not P21 retinas, compared to *Vegfa*^flox/flox^ retinas. No differences in expression of Cd93 in P7 retinas compared to P21 was noted regardless of Vegfa status. The expression of Bcl-2 was significantly decreased in retinas from P7 and P21 *Vegfa*^PC^ mice while Bim was significantly decreased in these mice only at P7 (Figure 9B). Bim and Bcl-2 expression was significantly increased in P21 retinas compared to P7 retinas regardless of *Vegfa* status. However, the increase in Bim expression in P21 *Vegfa*^flox/flox^ retinas compared to P7 retinas was not significant.

## 4. Discussion

VEGF production by pericytes is important for turning off signaling through both VEGFR2 and PDGFRβ by promoting their heterodimerization and stabilizing the pericyte vascular phenotype [24,54]. Endothelial cells also make VEGF, and its expression is essential for endothelial cell longevity. Deletion of VEGF in endothelial cells causes their loss as well as vascular degeneration of mature vessels without impacting early retinal vascularization [7]. However, the necessity of VEGF expression and its cell-autonomous activity in retinal pericytes remained unknown. Previous analysis of VEGF reporter mice demonstrated that retinal pericytes expressing VEGF appeared cell-associated, which led to the suggestion that VEGF could stabilize the newly forming vessels [11]. Similarly, here we showed Cre expression localized to pericytes, and not endothelial cells, by their abluminal surface localization on blood vessels and nuclear morphology, in *Vefga^PC^* mice. We also confirmed that Cre-expressing cells were positive for the pericyte-specific marker CD13. These results were consistent with the overall decreased VEGF expression noted in retinal RNA prepared from mature *Vefga^PC^* mice compared to control littermates.

Retinal vascularization proceeds after birth in the mouse with the superficial layer laid down in the first seven days of postnatal life (P7). Retinal endothelial cell sprouting and expansion during this process have been shown to be aided through expression of VEGFR1, a high-affinity receptor for VEGF on pericytes, restricting VEGF/VEGFR2 signaling on endothelial cells [34]. Pericyte deletion of VEGFR1 decreased retinal vessel outgrowth of developing vasculature without any impact on pruning [34]. This is consistent with the decreased expression of *Vegfr*1 noted in retinas from P7 *Vefga^PC^* mice compared to control littermates. The *Vefga^PC^* mice also showed decreased vessel outgrowth of the developing vasculature in the retinal superficial layer, corresponding with reduced sprouting tip cell numbers and pericyte densities compared with control littermates. However, it is unclear whether the noted abnormalities in P7 retinas from mice with pericyte deletion of *Vegfr*1 persists in older mice. We noted no obvious changes in endothelial cell number/density through development and remodeling nor in artery numbers in mature animals. Furthermore, the densities of pericytes and endothelial cells at six weeks of age were similar in *Vegfa*^PC^ mice compared with control littermates. Although the density of pericytes was modestly increased in six-month-old *Vegfa*^PC^ mice, no signs of abnormalities were noted in fundus and angiography images in 9-month-old *Vegfa*^PC^ or control mice.

VEGF enhances cell survival, in part due to its ability to stimulate Bcl-2 expression [17,18]. Bcl-2 deletion in pericytes (Bcl-2^PC^), utilizing the same *Pdgfrb*-Cre (Tg (*Pdgfrb*-Cre)*^45Vli^*), also diminished retinal vasculature superficial layer spreading, as we observed here in *Vefga*^PC^ mice. However, unlike in *Vegfa*^PC^ mice, we observed decreased pericyte and endothelial numbers before and after remodeling, a lack of central vessels in the optic papilla, and decreased numbers of retinal arteries and veins in Bcl-2^PC^ mice [37]. Lack of pericytes has a profound effect on vascular integrity [35,36]. However, lack of VEGF expression in pericytes does not have a profound impact. In the adult, the lack of VEGF expression in endothelial cells leads to destabilization of the mature retinal vasculature, with leaky and ghost vessels apparent. Even though pericytes express 4–6-fold higher levels of VEGF than endothelial cells, we did not observe destabilization of the mature retinal vascular in 6- or 9-month-old *Vefga*^PC^ mice. Thus, loss of pericyte VEGF expression delayed the migration of the superficial retinal vascular layer early, likely because of reduced pericyte recruitment. We also noted decreased pericyte numbers at 3 weeks of age. However, this did not have a long-lasting impact on the maturation of retinal vasculature. These observations are consistent with increased levels of Cxcl11/Cxcr3 and an early decrease in Cd93 in retinas from *Vegfa*^PC^ mice. The expression of the proinflammatory mediator *Cxcl*1 was also significantly increased in P21 *Vegfa*^PC^ compared to *Vegfa*^flox/flox^ P7 or P21 retinas. 

Cxcl11 and Cxcr3 are expressed by both endothelial cells and pericytes, and their coordinated activities in these cells are important for formation of new blood vessels and their maturation and stabilization. Increased signaling through the Cxcl11/Cxcr3 axis in endothelial cells results in decreased sprouting and increased pericyte recruitment, while enhanced signaling in pericytes enhances their recruitment to the newly formed sprouts by endothelial cells and suppression of new vessel formation [55,56]. How these activities are coordinated during vascular development and neovascularization and, more specifically, the role of VEGF/VEGFR1/VEGFR2 signaling in endothelial cells and pericytes in impacting these activities need further investigation. Similarly, the interaction of endothelial cells with pericytes promotes the expression of Mmrn2. Interaction of Cd93 with Mmrn2 results in its stabilization and promotion of angiogenesis and vascular maturation through modulation of β1 integrin activity [48,51,57]. In fact, the binding of Cd93 and Mmrn2 promotes choroidal sprouting ex vivo and choroidal neovascularization in vivo [50,58]. Thus, decreased levels of Cd93 in retinas from *Vegfa*^PC^ mice are consistent with reduced expansion of the developing superficial vascular layer.

We previously showed that pericytes play a vital role in remodeling of the vascular basement membrane and extravasation of neutrophils during inflammation [59]. *Cxcl*1 is produced by both endothelial cells and pericytes in response to inflammatory mediators including TNF, and its signaling through Cxcr2 supports luminal and sub-endothelial cell neutrophil crawling [60]. *Cxcl*1 expression was recently shown to be a mediator of neutrophil recruitment and the pathogenesis of diabetic retinopathy, perhaps through pericyte-mediated alterations in retinal vessel barrier function [61], which may require IL-17a [62]. How paracrine and autocrine VEGF signals in retinal endothelial cells and pericytes regulate the expression and interactions of these proteins represents a subject for future investigation.

Pericytes are vulnerable to adverse conditions including hyperoxia and high glucose levels, leading to retinopathy of prematurity (ROP) and diabetic retinopathy. During OIR, the murine equivalent of ROP, the retinal VEGF expression decreases in hyperoxia, and when the animal is brought to room air, the retina becomes ischemic, inducing vascular loss around the optic nerve, causing VEGF levels to increase and aberrant tortuous leaky new vessels to form. Lack of pericyte VEGF expression did not impact the amount of vessel obliteration or neovascularization in the OIR model, similar to what we observed previously when Bcl-2 expression was lacking in pericytes using the same Cre-transgenic line. This is an interesting turn of events, given that the lack of Bcl-2 expression decreases vascular density while the lack of VEGF had minimal impact. Thus, pericyte signaling through the VEGF/Bcl-2 axis does not impact pathogenic changes noted during OIR.

VEGF interacts with various receptors and impacts the expression of many genes with important roles in the regulation of angiogenesis and vascular function. Here we examined whether the expression of VEGF in pericytes impacts the expression of various angio- and inflammatory regulatory genes including various forms of Vegf and its receptors in retinas from *Vegfa*^flox/flox^ and *Vegfa*^PC^. We showed that Vegfa and Vegfr2 were more predominantly expressed in the retina compared to other isoforms of Vegf and Vegf receptors examined here. We noted that Vegfa expression was significantly decreased in retinas from P21 *Vegfa*^PC^ mice as expected. Vegfr2 expression was also lower in retinas from P7 and P21 *Vegfa*^PC^ mice, but this was not significant. The significant decrease in Vegfa level was consistent with a significant decrease in the number of pericytes and Vegfr1 expression in P21 retinas from *Vegfa*^PC^ mice. We also noted a decrease in the level of *Pdgfb* in retinas from P7 and P21 *Vegfa*^PC^ mice compared to their *Vegfa*^flox/flox^ littermates that was significant at P21 even though Pdgfrb expression was not impacted by the lack of Vegfa in pericytes. 

Production of angiopoietins (Angpts) and their interaction with their receptor Tek/Tie2 impacts angiogenic activities through the modulation of endothelial and pericyte interactions and vascular functions [63]. Angpt1 enhances and establishes the interaction between endothelial cells and pericytes in newly formed vessels. However, Angpt2 antagonizes the activity of Angpt1 by promoting pericyte migration and enhancing angiogenesis by VEGF. In contrast, Angpt2 promotes vessel degeneration in the absence of VEGF [64]. The level of Angpt1 was significantly higher in retinas from P7 and P21 *Vegfa*^PC^ mice compared to their *Vegfa*^flox/flox^ littermates. However, the significant increase in Angpt1 in P21 mouse retinas compared to P7 was independent of Vegfa expression in pericytes. Similarly, the expression of *Angpt2* increased in retinas from P7 and P21 *Vegfa*^PC^ mice compared to their *Vegfa*^flox/flox^ littermates, which was significant at P21. In contrast to *Angpt*1, the level of Angpt2 is significantly lower in P21 retinas compared to P7 retinas, independent of Vegfa expression in pericytes. The expression of Tak/Tie2 similarly increased in P7 and P21 retinas from *Vegfa*^PC^ compared to their *Vegfa*^flox/flox^ littermates, even though we did observe a modest but significant increase in Tak/Tie2 expression at P21 in *Vegfa*^PC^ mice. The expression of Tak/Tie2 was significantly increased in P21 retinas compared to P7 retinas regardless of Vegfa status in pericytes. These studies are consistent with the minimal impact of pericyte Vegfa expression on retinal vascular integrity and function noted here.

The increased expression of αSMA in pericytes is an indication of their activation that is likely impacted by changes in VEGF levels. Here we noted a significant increase in αSMA levels in retinas from P7 *Vegfa*^PC^ mice compared to their *Vegfa*^flox/flox^ littermates. This is consistent with decreased number of pericytes in retinas from P7 *Vegfa*^PC^ mice. No differences were noted in the level of αSMA in retinas from P21 *Vegfa*^PC^ mice compared to their *Vegfa*^flox/flox^ littermates. The level of αSMA was significantly increased at P21 compared to P7, regardless of VEGF status. This is consistent with the lack of vascular abnormalities noted in P21 *Vegfa*^PC^ mice. Although the expression of the other pericyte marker, Cspg4/Ng2, was similar in P7 and P21 retinas from *Vegfa*^PC^ compared to their *Vegfa*^flox/flox^ littermates, there was a significant increase in Cspg4/Ng2 expression at P21 compared to P7 that was independent of pericyte *Vegfa* status.

The outer retina is nourished by the choroidal vasculature. Disturbances in this vasculature are noted in disease states including nAMD. Pericytes act as vascular stabilizers, with their physical contact with endothelial cells not only stabilizing the vasculature but also preventing vascular leakage. Pericytes produce survival factors such as VEGF, which in turn enhances Bcl-2 preventing apoptosis [17,18]. Here we showed that Bcl-2 expression decreased in the retina when VEGF was lost in pericytes. We had previously shown that loss of Bcl-2 in pericytes decreased both pericyte and endothelial cell numbers with retinas having fewer arteries and veins [37]. Unlike Bcl-2^PC^ mice, here we only observed decreased retinal pericyte numbers in P21 *Vegfa*^PC^ mice with no change in artery numbers. Delayed advancement of the superficial retinal vascular layer and decreased tip cell number were also observed in *Vegfa*^PC^ mice, similarly to what we previously noted in Bcl-2^PC^ mice [37]. Also, both *Vegfa*^PC^ and Bcl-2^PC^ mice did not demonstrate any significant alteration in the degree of pathologic neovascularization with OIR compared to control littermates. When pericytes lacked VEGF expression, mice demonstrated similar levels of CNV, suggesting that pericyte VEGF expression is not essential for ocular pathologic neovascularization. This contrasts with Bcl-2 expression in pericytes, which, when lost, decreases CNV [37].

The absence of a marked effect on retinal vascular development and neovascularization may be due to compensatory regulation of VEGF and/or its receptors in other ocular cell types. In our study, we detected a two-fold reduction in VEGF expression in *Vegfa*^PC^ retinas. A limitation of our study is that the protein levels of VEGF and of the other regulatory genes whose expression we examined were not assessed. Notably, loss of VEGF expression in pericytes did not alter either retinal or choroidal neovascularization. This deficiency, however, may influence the regression of newly formed vessels, a process that proceeds normally with these models and deserves consideration. Nevertheless, our findings indicate that pericyte-derived VEGF is not essential for retinal vascular development or maturation. Given that choroidal vessels are also invested with pericytes, it will be important to investigate whether pericyte-derived VEGF contributes to normal choroidal vascularization and responses to oxidative and inflammatory stress, particularly in the context of choroidal vascular and outer retinal degenerations associated with dry AMD.

## 5. Conclusions

Paracrine and autocrine VEGF signaling in endothelial cells and pericytes play vital roles during proper tissue vascularization, and their alterations contribute to the pathophysiology of many diseases including retinopathy of prematurity, diabetic retinopathy, and AMD. The delineation of the underlying cellular and molecular mechanisms involved will advance our knowledge regarding disease pathogenesis and the development of novel and effective treatments. However, our data collectively demonstrate a limited role for pericyte VEGF expression in normal and pathological ocular vascularization. 

## Figures and Tables

**Table 2:** Retinal vascular cell numbers.

Cell Type	Age	*Vegfa* ^flox/flox^	*Vegfa* ^PC^
Pericyte	3 weeks	27.46 ± 1.61	21.17 ± 0.76 **
Endothelial Cell	3 weeks	118.50 ± 4.51	115.60 ± 2.26
Pericyte	6 weeks	20.43 ± 0.61	19.67 ± 1.62
Endothelial Cell	6 weeks	102.40 ± 2.32	100.00 ± 5.27
Pericyte	6 months	18.57 ± 0.78	22.13 ± 1.00 **
Endothelial Cell	6 months	94.86 ± 2.30	94.06 ± 1.74

Number of cells per high-power field (×400, 100 µm^2^). The *p* values were calculated by comparing samples from *Vegfa*^flox/flox^ to *Vegfa*^PC^ at the ages noted. ** *p* < 0.01.

**Table 3:** Gene expression changes.

Genes	P7 vs. P7 Cre− vs. Cre+	P21 vs. P21 Cre− vs. Cre+	P7 vs. P21 Cre−	P7 vs. P21 Cre+
*Vegfa*	4% ^#^	26% *	38% ***	22%
*Vegfb*	21% *	7%	48% **	56% **
*Vegfc*	ND	7% *	32% ***	37% ***
*Vegfr1*	50% ***	22% *	87% **	92% **
*Vegfr2*	17%	14%	11%	13%
*Vegfr3*	11%	11%	50% **	50% **
*Pdgfb*	30%	23% **	15%	23% **
*Pdgfrb*	11%	8%	63% ***	63% ***
*Angpt1*	20% *	18% **	25% *	24% **
*Angpt2*	7%	15% **	50% **	45% **
*Tek/Tie2*	14%	5% *	50% ***	45% **
*Acta2/αSma*	45% ***	ND	68% ***	42% **
*Cspg4/Ng2*	4%	ND	45% *	47% *
*Mmrn2*	14%	4%	50% *	43% **
*Cd93*	30% **	ND	ND	ND
*Cxcl1*	15%	34% **	33% *	48% **
*Cxcl11*	43% *	36% *	47% *	36% *
*Cxcr3*	31% **	30% *	47% *	36% *
*Bcl-2*	39% *	11%	71% ***	80% **
*Bim*	20% *	1%	16%	32% **

^#^ Red denotes a decrease. Black denotes an increase. ND: no difference (* *p* < 0.05. ** *p* < 0.01. *** *p* < 0.001).

## Data Availability

The original contributions presented in this study are included in the article. Further inquiries can be directed to the corresponding authors.
